# The long-term prediction of return to work following serious accidental injuries: A follow up study

**DOI:** 10.1186/1471-244X-11-53

**Published:** 2011-04-06

**Authors:** Urs Hepp, Hanspeter Moergeli, Stefan Buchi, Helke Bruchhaus-Steinert, Tom Sensky, Ulrich Schnyder

**Affiliations:** 1 Psychiatrische Dienste Aargau AG, Baden, Switzerland; 2 Department of Psychiatry, University Hospital Zurich, Zurich, Switzerland; 3 Privatklinik Hohenegg, Meilen, Switzerland; 4 Institute for Ecological Systemic Therapy, Zurich, Switzerland; 5 Division of Neurosciences and Psychological Medicine, Imperial College School of Medicine, West Middlesex Hospital, Isleworth, Middlesex, UK

## Abstract

**Background:**

Considerable indirect costs are incurred by time taken off work following accidental injuries. The aim of this study was to predict return to work following serious accidental injuries.

**Method:**

121 severely injured patients were included in the study. Complete follow-up data were available for 85 patients. Two weeks post trauma (T1), patients rated their appraisal of the injury severity and their ability to cope with the injury and its job-related consequences. Time off work was assessed at one (T2) and three years (T3) post accident. The main outcome was the number of days of sick leave taken due to the accidental injury.

**Results:**

The patients' appraisals a) of the injury severity and b) of their coping abilities regarding the accidental injury and its job-related consequences were significant predictors of the number of sick-leave days taken. Injury severity (ISS), type of accident, age and gender did not contribute significantly to the prediction.

**Conclusions:**

Return to work in the long term is best predicted by the patients' own appraisal of both their injury severity and the ability to cope with the accidental injury.

## Background

Sick-leave following accidental injuries incurs considerable indirect costs and although the amount of time lost from work is one of the most important measures of functional outcome of injuries [1], there are few studies on return to work after severe accidental injuries [2-8]. Return to work is not only predicted by injury related factors. Job related factors [2,9,10], socioeconomic factors [2,4,6], psychological distress [6,8], causal attribution [11] and compensation eligibility [7] are predictive factors for return to work. How patients' expectations of recovery affect their health outcomes is insufficiently researched [12]. Patients returning to work after injury had stronger internal health beliefs, i.e. they believed they had an influence on their health and experienced themselves as powerful [4]. The influence of appraisal on the process of coping with stress has been the scope of a large body of research of Lazarus [13]. In a prospective long term study on accidentally injured patients we found the patients' own self-reported appraisal of injury severity and their ability to cope with the accidental injury, and its job-related consequences, predicting time off work 12 months after the accident [5]. The aim of this study was to predict return to work three years post accident. Our hypothesis was that the patients' appraisal is still predictive for return to work.

## Materials and methods

### Subjects

Participants were recruited from the Division of Trauma Surgery, Department of Surgery at Zurich University Hospital. All the patients qualifying for the study had sustained accidental injuries that caused a life-threatening or critical condition requiring their referral to the intensive care unit (ICU). Participants had to meet the following criteria to be included in the study: age between 18-70 years; sufficient proficiency in the German language to participate in the interview and to complete the questionnaires; a clinical condition allowing participation in an extensive clinical interview within one month of the accident. Furthermore, an Injury Severity Score (ISS) [14] of 10 or more and a Glasgow Coma Scale score (GCS) [15] of 9 or more were required, which allowed us to collect a sample of severely injured participants without severe traumatic brain injury. Patients were excluded if they were suffering from any serious somatic illness; had been under treatment for any mental disorder immediately prior to the accident; had shown marked clinical signs or symptoms of mental disorders that were obviously unrelated to the accidental injury; had been referred due to attempted suicide or were victims of physical violence which had caused their injuries.

All patients referred to the ICU were consecutively screened over a period of 18 months. 16 patients were excluded due to the presence of pre-existing psychiatric morbidity. 135 patients were eligible for the study; 14 refused to participate. Written informed consent was obtained from 121 patients. The initial interview was performed an average of 13 days (SD 7, min 3, max 29 days) after the accident (T1). Follow-up interviews were conducted 12 months (T2) and 36 months (T3) post accident.

A total of 90 patients participated in all three interviews. Comparison between these patients (N = 90) and the 14 who refused to participate did not reveal significant differences with regard to sex, age, ISS and GCS scores. Work-related accidents were significantly more frequent among the patients who refused to participate (non-participants: 7; 50%, sample: 13; 14.4%; Fisher's exact test, P < 0.01). There were no significant differences between the 31 drop-outs and the 90 patients who participated in all three interviews with regard to sociodemographic variables and variables related to the accidental injury, except for marital status (drop-outs: 21 single, 9 married, 1 divorced; sample: 34 single, 43 married, 13 divorced. Chi-square = 8.95, df = 2, P < 0.05) and appraisal of injury severity (drop-outs: 3.9 (SD = 1.0); sample: 4.3 (SD = 0.8), t = 2.01, df = 113, P < 0.05). Specifically, no differences were found for the following scales: Injury Severity Score (ISS), Glasgow Coma Scale (GCS), Impact of Event Scale (IES), appraisal of coping abilities. Among the 31 drop-outs we had 12 months (T2) data regarding time off work from 15 patients. 12 months time off work did not differ between these 15 patients and the final sample (15 drop-outs: 180 days (SD = 131); sample: 206 days (SD = 120), t = 0.76, df = 98, n.s.). Finally, 5 patients who were either retired, homemakers or who had given incomplete data had to be excluded from the analysis regarding fitness for work. Therefore, complete follow-up data were available for 85 patients (50 road traffic, 13 workplace, 5 household, 17 leisure-time accidents). 67 (79%) were male, the mean age was 37.7 (SD = 12.4) years. The mean ISS was 22.2 (SD = 10.3). More detailed information with regard to clinical parameters of the sample has been published in a previous article [16].

### Measures

The Injury Severity Score [14] permits an evaluation of the severity of injuries by a trauma surgeon: Each part or area of the body affected is given a score (1 = minimum, 6 = fatal injury; if the score is 6 in one area, the ISS score is assigned to 75). The scores for the three most severely injured areas of the body are squared and then summed, producing maximum score of 75. Patients with a score of 10 or more are generally considered severely injured. The Glasgow Coma Scale [15] is an observer-rated scale for the clinical appraisal of the gravity of coma after injury to the skull and brain. Patients with severe traumatic brain injuries generally have a score under 9. ISS and GCS were routinely assessed by the surgeons immediately after admission to the emergency room.

In the semi-structured interviews two weeks post trauma (T1), socio-demographic data, including a detailed work record and information about the accidents were collected. The patients rated their appraisal of the injury severity on a Likert scale ranging from "1 = very slight" to "5 = very severe". They also rated their ability to cope with the accidental injury and its job-related consequences on a Likert scale ranging from "1 = very poor" to "5 = very good" [5]. Time off work, assessed at one (T2) and three years (T3) post-accident, was defined as the number of days of sick leave taken due to the accidental injury and its consequences including time of hospitalization.

Posttraumatic psychological symptoms were assessed by using the Impact of Event Scale (IES) [17], a 15 item self-rating questionnaire comprising two subscales (intrusion and avoidance) with high reliability and validity as a screening instrument for posttraumatic stress disorder [18]. In the final sample internal consistency was high for the intrusion subscale (Cronbach's α = .90) and moderate for the avoidance subscale (Cronbach's α = .76).

All the interviews and assessments at T1 and T2 were conducted by an experienced medical doctor. The assessment at T3 was performed by another experienced medical doctor.

### Statistical analyses

Time taken off work was calculated as the number of days of leave taken from the time of the injury (including time in hospital), with a week off work equaling seven days of leave. Where subjects who had previously been full-time employees returned to work on a part-time basis, the days on which they worked less were added to the total days of leave on a pro rata basis.

For the prediction of the number of sick leave days taken at three years follow up, hierarchical linear multiple regression analyses were performed. They allowed for highlighting the relevance of patient's appraisal among the selected potential predictor variables. To enter the type of accident (road traffic, workplace, household, or leisure-time accidents) as a predictor into the multiple regression analysis, this categorical variable was converted into a set of three new variables so that a deviation contrast resulted. Accordingly, the effect of each accident category was compared to the mean effect of all accident categories. Since there was one new variable for each degree of freedom, one accident category (household) had to be omitted in the regression analysis. In the final regression model including all potential predictors multicollinearity was low (tolerance >0.6) and the distribution of regression standardized residuals was normal (Kolmogorov-Smirnov Z = 1.31, n.s.). Group comparisons of dimensional variables were performed with t-tests.

### Ethical approval

Ethical approval was granted by the Institutional Review Board of the Canton of Zurich. Written informed consent was obtained from all the patients.

## Results

Socio-demographic characteristics and characteristics related to the accidental injury of the sample are presented in Table 1 and 2.

**Table 1:** Sociodemographic characteristics of severely injured accident victims (N = 85)

*Variable*	*N*	*%*
*Age: *Mean years ± standard deviation	37.7 ± 12.4	
*Sex:*		
Male	67	(78.8%)
Female	18	(21.2%)
*Marital status:*		
Single	34	(40.0%)
Married	38	(44.7%)
Divorced	13	(15.3%)
*Living arrangements:*		
Alone	17	(20.0%)
With others (family, partner, friends)	68	(80.0%)
*Maximum educational level:*		
No education	2	(2.4%)
Obligatory school	10	(11.8%)
Apprenticeship	48	(56.5%)
College	3	(3.5%)
Technical or commercial college	17	(20.0%)
University	5	(5.9%)
*Employment status:*		
Paid work (full- or part-time)	79	(92.9%)
Student	6	(7.1%)

**Table 2:** Accidental injury related characteristics of severely injured accident victims (N = 85)

*Variable*	*Mean*	*SD*	*Minimum*	*Maximum*
Injury Severity Score	22.2	10.3	10	51
Glasgow Coma Scale	14.4	1.6	9	15
Length of stay (days) at the ICU	5.8	5.2	1	26
Length of stay (days) at the University Hospital ^a^	33.0	33.4	1	220
Length of stay (days) at the University Hospital and Rehabilitation ^a^	73.3	79.2	4	365
Time off work ^a^	371.1	359.4	25	1095

^a ^Subsumes the row above it

Table 3 shows the bivariate correlations of all variables included in regression analyses. Time off work correlated significantly with IES intrusion, the patients' own appraisals of both their injury severity and their coping abilities. Higher age was associated with lower injury severity as well as fewer road traffic accidents. Work related accidents were more frequent in males and correlated with higher IES intrusion scores. The patients' appraisals of the injury severity and of their coping abilities correlated with IES intrusion. However, the two appraisals variables did not correlate with each other nor did they correlate significantly with injury severity.

**Table 3:** Bivariate correlations between potential predictor variables^a ^to each other and to the dependent variable time off work due to the accidental injury^b^

Variable	TOW	ISS	SEX	AGE	TRAFF	SPORT	WORK	IESIN	AIS
ISS	0.04								
SEX	0.06	-0.12							
AGE	0.16	-0.24*	0.00						
TRAFF	-0.07	0.09	0.02	-0.41***					
SPORT	-0.16	0.04	-0.03	0.03	-0.05				
WORK	-0.03	-0.01	-0.24*	-0.07	0.03	0.21			
IESIN	0.35***	-0.12	0.01	0.17	-0.07	-0.03	0.31**		
AIS	0.34***	-0.06	-0.09	0.17	-0.04	0.06	0.02	0.35***	
ACA	-0.36***	-0.08	-0.11	0.11	-0.04	0.07	-0.03	-0.39***	-0.13

Pearson correlation coefficients, N = 85

^a ^Assessed 3-29 days after the accident

^b ^Assessed 3 years after the accident

*p ≤ 0.05, **p ≤ 0.01, ***p ≤ 0.001

Variable   Explanation

TOW   Time off work (days) due to the accidental injury

ISS   Injury Severity Score

SEX   Gender (1 = male, 2 = female)

AGE   Age

TRAFF   Type of accident: traffic

SPORT   Type of accident: sports or leisure time

WORK   Type of accident: workplace

IESIN   Impact of Event Scale, Intrusion subscale

AIS   Appraisal of injury severity

ACA   Appraisal of coping abilities

In a simultaneous regression analyses the variables injury severity, sex, age, type of accident (road traffic, workplace, or leisure-time accidents), and IES intrusion were entered as potential predictors of time off work. In combination, these predictors explained 18% of the variance of time off work (F = 2.34, df = 7;77, p < 0.05). When in a series of hierarchical regressions each of these predictors was examined when added last to this first set, only IES intrusion added unique variance (11.2%, F = 10.5, df = 1;77, p < 0.01). These five variables were then treated as the first set added in hierarchical regressions focusing on two additional predictors, patients' appraisals of the injury severity and of their coping abilities. These two variables were entered in the second step accounting for an additional 11.2% of the variance of the time off work three years post accident (F change = 5.87, df = 2;75, p < 0.01). Self reported appraisal of the injury severity added 5.6% (F change = 5.56, df = 1;76, p < 0.05), and self reported appraisal of their coping abilities added 5.8% (F change = 5.73, df = 1;76, p < 0.05). Finally, each of the seven predictors in Table 4 was evaluated for unique variance contributed with the other six predictors already in the model. The two appraisals variables remained significant, whereas the severity of the injury (ISS), type of accident, IES intrusion, age and gender did not contribute significantly to the prediction.

**Table 4:** Prediction of time off work

	*1 year*		*3 years*	
*Predictor variable*	*Beta*	*p*	*Beta*	*p*
Injury Severity Score (ISS)	.21	**<.05**	.10	n.s.
Female gender	.08	n.s.	.05	n.s.
Age	.16	n.s.	.13	n.s.
Type of accident: traffic	.03	n.s.	-.03	n.s.
workplace	.03	n.s.	-.04	n.s.
sports/leisure	-.22	**<.05**	-.15	n.s.
IES Intrusion subscale	.12	n.s.	.15	n.s.
Appraisal of injury severity	.35	**<.001**	.25	**<.05**
Appraisal of coping abilities	-.23	**<.05**	-.27	**<.05**

Multiple Regression: 1-year: N = 100, R = .60, R^2 ^= .36, p < .001

Multiple Regression: 3-year: N = 85, R = .54, R^2 ^= .29, p < .01

**Figure 1:**
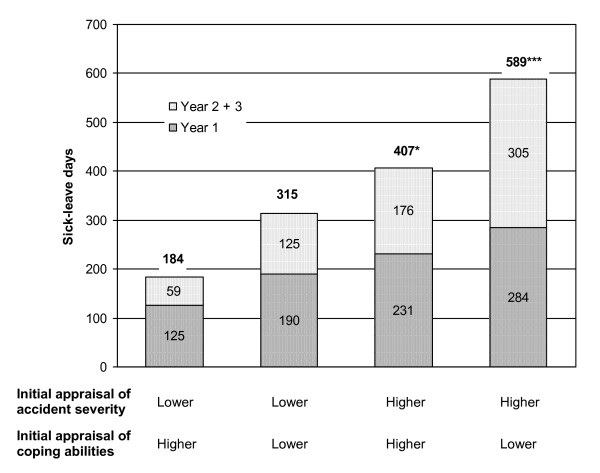
**Sick-leave days of accident victims depending on appraisals of accident severity and coping abilities (N = 85, n = 19 to 23 per group)**. Comparison of the group „lower appraisal of accident severity and higher appraisal of coping abilities" with the three other groups: *p≤.05, *** p≤.001

In order to visualize the effects of appraisals on sick-leave days taken the sample was divided into four groups based on the median values for appraisal of injury severity and of coping abilities (Figure 1). The median for both variables was 4 Likert points. Patients with values = 5 were grouped as 'higher' and patients with values < = 4 were grouped as 'lower'. Regarding the two groups of particular interest, namely patients who assessed the injury severity as higher and their coping abilities as lower compared with patients who estimated the injury severity as lower and their coping abilities as higher, there were three times as many sick-leave days for the former group (t = 4.22, df = 35.4, p <. 001). In addition, the increase in sick-leave days in year two and three post accident showed a significant difference between these two groups (t = 3.03, df = 35.5, p <. 01).

## Discussion

Return to work is undoubtedly one of the most significant outcome measures after severe accidental injury. Indirect costs associated with injury exceed direct costs of treatment and sick-leave following accidental injury is a major contributor to the total burden of health care costs [2,19]. The relevance of psychosocial and subjective factors for a successful return to work is increasingly recognized [4,8,12,20] and from chronic back pain patients it is known that the longer individuals refrain from work, the lower the probability of returning to work [21,22].

One year after accidental injury we could show that patients' self-reported appraisal of injury severity and of patients' ability to cope with the accidental injury and its job-related consequences were the strongest predictors of return to work [5]. Injury severity and the type of accident which were also predictors at one year, no longer contributed to the prediction at the follow-up. At three years only patients' appraisal of injury severity and of patients' ability to cope remained predictive - independent of each other and of objective injury severity measured by the ISS as bivariate correlations show.

We anticipated that the patients' appraisal of injury severity and their own ability to cope with the accidental injury and its job-related consequences would still contribute to the prediction of time off work at three years but we anticipated less impact. In fact, the difference between the 4 groups actually increased at three years. In the first year medical treatment and rehabilitation contributed significantly to days off work (Table 2), whereas in the second and third year after the injury, factors not related to the accidental injury gained influence.

These results are in line with Lazarus' theories on stress, appraisal and coping [13,23]. Lazarus emphasized the significance of the primary and secondary appraisal of a stressful situation. In the primary appraisal the situation can be judged as harmful, as a threat or as a challenge. The same situation can be appraised differently by different individuals. The secondary appraisal is the person's judgment of his/her ability to cope with a situation and this depends on the person's individual coping strategies. When a stressful situation is appraised as controllable by action, problem-focused coping will predominate, whereas where a situation is viewed as refractory to change, emotion-focused coping is more likely to predominate. Coping is increasingly viewed as a process rather than a style and can change over time in accordance with the situational context [13,24].

The fact that injury severity was no longer predictive at the three year follow-up needs further explanation. Findings from other studies on this point are not consistent. Time off work in severely injured accident victims correlated with physical impairment in some studies [2,4] whereas there was no correlation with injury severity in others [6,8,25]. In work related low back injuries and hand trauma, objective measures of physical impairment correlated with return to work but were less important than psychosocial factors [7,9]. Contrary to our findings, Soberg et al. found that injury severity after severe multiple injuries was higher in the non-return to work group at 2-years follow-up but not at one year [4]. The one year results were interpreted so that the contribution of time of hospitalization and rehabilitation was more important in the first year. In our sample we also observed that in the first year post accident the length of hospitalization contributed significantly to the time off work, whereas in the following years this was no longer relevant. An important reason for the divergent results could be the exclusion of severe brain injury in our sample. 36% of the patients in the Soberg study had sustained a head/neck injury, 18% had a spinal cord injury. It might be hypothesized that in these types of injury the injury severity has more impact on the functional outcome and on return to work than in non-neurologic injuries. McKenzie et al. [2] excluded patients with major neurologic injury and still found a correlation between injury severity and return to work, but this correlation was weak. Impairment at hospital discharge and in the follow up assessments better predicted return to work than the initial injury severity.

The fact that in our study injury severity was no longer predictive of the time off work may be partially explained by an overall high ISS score in the study sample. All the participants in the study were severely injured and therefore did not fully represent the whole spectrum of victims of accidental injuries.

Advanced age, which is generally regarded as a risk factor for non-return to work [2,9,25], was not predictive in our study. This result is in accordance with the Soberg study [4]. Our sample's relatively low proportion of workplace accidents, where age might have a greater impact on outcome, could be one reason for this finding [9].

The general impact of patients' personal expectations and health beliefs on health outcome is increasingly recognized. A review found correlations between positive expectations and better health outcome for different medical conditions [12]. After major limb trauma, one of the most important predictors of rate of return to work was work self efficacy or the patients' belief that they are able to return to work [26]. Patients who sustained multiple injuries and returned to work two years after the accidental injury scored higher on internal health beliefs, i.e. they believed they had some influence on their own wellbeing. Patients who did not return to work scored higher on external health beliefs, i.e. they believed their health was dependent on "powerful others" or factors beyond their influence [4]. In patients who sustained traumatic injury patients' characteristics like higher level of education, high levels of social support, job stability, white collar employment and employment in jobs with low physical demands and good benefits were associated with higher rates of return to work [2]. All these factors can have an influence on patients' appraisal of their coping abilities. In low-back pain patients their prediction of outcome and return to work is of high prognostic value [22,27], and following myocardial infarction patients' initial positive beliefs concerning their illness favored return to work [28].

Mayou et al. found in a three-year follow-up after motor vehicle accidents that psychological factors, persistent medical and financial problems and ongoing litigation were important predictors of chronic posttraumatic stress disorder whereas psychiatric outcome and pain were no longer related to the initial injury severity. One out of three patients in this sample developed a psychiatric complication [29,30]. Despite these findings, psychiatric or psychological assessment is uncommon in victims of accidental injuries and the main focus is still on the pure somatic treatment. The poor outcome of many accident victims, independent of the objective severity of the injury, confirms the importance of early psychological assessment and, where needed, treatment and the provision of practical advice and information [29]. Michaels et al. state that psychological morbidity following injury impedes return to work. Despite the observation of a gap between physical outcome and return to work, the management of psychological and social consequences of injury is still neglected [6].

Some limitations of this study have to be addressed. The sample included only severely injured accident victims and whilst the homogeneity of the sample helps in the interpretation of the results, it also increases the likelihood that the results may not be generalized to apply to patients with less severe injuries.

We excluded patients with pre-existing somatic and psychiatric morbidity in order to achieve a sample as homogenous as possible and to reduce the possibility of the outcome being influenced by factors other than the accidental injury. By excluding patients with pre-existing somatic and psychiatric morbidity we possibly excluded patients who were at higher risk for sick-leave following accidental injury. Knowledge of the German language as an inclusion criterion might have led to the exclusion of participants who were less well socially integrated, a risk factor for work disability. It can be hypothesized that insufficient proficiency in the German language would have resulted in greater difficulties in dealing with the consequences of accidental injuries and, therefore, longer time off work. The question remains whether such an outcome would be mediated by the patients' appraisals or other factors related to the knowledge of the German language, such as the level of education. In a follow up study non-German speaking participants were included and interviewed using interpreters and translated questionnaires. There was no significant difference between German speaking and non-German speaking participants with regard to PTSD symptoms [31].

There were 31 drop-outs from T1 to T3. At the 12 months follow up (T2) data from 15 patients who did not participate at the 3-year follow-up (T3) was available. However, these 15 participants did not significantly differ from the final sample. There was lower appraisal of the injury severity and a non-significant trend to less time off work in these drop-outs. Patients with a higher risk for longer sick-leave were, therefore, well represented in the final sample and the drop-outs probably did not affect the results substantially.

The number of days off work was assessed in the interviews by patients own self-rating. Strict data privacy protection laws in Switzerland prevent the use of health insurance companies' data for the purpose of research projects. That data, of course, would have been more reliable.

## Conclusions

The appraisal of injury severity and of patients' ability to cope with the accidental injury and its job-related consequences predicted the time of sick-leave related to the accidental injury even three years post accident, independent of injury severity. It would appear that by asking two simple questions about patients' appraisals, it is possible to obtain relevant prognostic information regarding long-term return to work. A comprehensive treatment after accidental injuries should routinely be accompanied by a psychosocial assessment including information and practical advice.

## Competing interests

The authors declare that they have no competing interests.

## Authors' contributions

US and HM designed the study. HM and HBS were involved in the data collection. HM performed the statistical analyses. UH, HM, US, SB, TS were involved in the interpretation of the data. UH and HM drafted the manuscript. US, TS, SB reviewed the manuscript several times. All authors have read and approved the final version of the manuscript.

## Pre-publication history

The pre-publication history for this paper can be accessed here:

http://www.biomedcentral.com/1471-244X/11/53/prepub
